# The efficacy of a rubber bristles interdental cleaner on parameters of oral soft tissue health‐a systematic review‐

**DOI:** 10.1111/idh.12492

**Published:** 2021-05-29

**Authors:** Fridus van der Weijden, Dagmar Else Slot, Eveline van der Sluijs, Nienke Lisette Hennequin‐Hoenderdos

**Affiliations:** ^1^ Department of Periodontology Academic Center for Dentistry Amsterdam (ACTA) University of Amsterdam and Vrije Universiteit Amsterdam The Netherlands; ^2^ Scienfitic Office Meander Medical Centre Amersfoort The Netherlands

**Keywords:** dental plaque, gingival health, rubber bristles interdental cleaner, systematic review

## Abstract

**Aim:**

This study aimed to establish the efficacy of a rubber bristles interdental cleaner (RBIC) as an adjunct to toothbrushing (TB) compared to that of the adjuvant use of other interdental cleaning devices and TB alone on plaque and gingivitis parameters. Additionally, the safety aspects and panellists’ appreciation were evaluated.

**Materials and Methods:**

Databases were searched for randomized controlled clinical trials (RCTs) evaluating plaque (PI), bleeding (BS), and gingival index (GI) scores, safety assessments, and participants’ appreciation. Extracted data were summarized in a descriptive and, if possible, a meta‐analysis.

**Results:**

The search retrieved 142 unique papers; six studies with 10 comparisons were included in a descriptive analysis. Five RCTs compared RBICs with interdental brushes (IDBs), four with dental floss (DF) and one with manual TB only. No comparisons to wood sticks were retrieved. Using an RBIC resulted in no difference in plaque scores compared to DF and IDBs. For overall bleeding scores, no difference was found. Two studies analysing the accessible sites separately found RBICs to be more favourable than DF and IDBs. Conversely, one study evaluating the efficacy of RBICs compared to IDBs, according to the GI scores, showed that IDBs achieved significantly greater reduction. Moreover, RBICs caused fewer gingival abrasions and were preferred by the study participants.

**Conclusion:**

Based on a descriptive and a meta‐analysis of the available literature, it is synthesized that in gingivitis patients, a weak to very weak certainty exists that a RBIC is indicated for gingivitis and plaque reduction. The evidence supports user safety and participants’ preferences.

## 1 INTRODUCTION

Toothbrushing is the most common method of mechanical biofilm removal. With a good design and careful technique, a toothbrush can clean the embrasure, the open and easily accessible area between teeth. However, toothbrush bristles cannot properly penetrate under the contact point and efficiently reach into the interdental areas, resulting in parts of the teeth remaining uncleaned. A study by Lang and co‐workers found that plaque formation starts in the interdental spaces of molars and premolars and subsequently progresses in the interdental spaces of the anterior teeth.^1^ A further study demonstrated that interdental surfaces are the most difficult to clean.^2^ Thus, the interdental space constitutes a predilection site for diseases such as caries and periodontal disease. Moreover, recent studies provide convincing data supporting the use of interdental cleaning devices for promoting good oral health outcomes, particularly for secondary prevention. They found that interdental cleaning is associated with less periodontal disease, fewer coronal and interproximal caries, and fewer missing teeth.^3^, ^4^

Various products and methods have been introduced over the counter for interdental cleaning, such as dental floss, wood sticks, oral irrigators and interdental brushes (IDBs). Thus, oral care professionals have more than one choice when providing recommendations to their patients. Despite the wide range of marketed oral hygiene products, much of the dental literature remains somewhat equivocal on the relative benefits of different interdental oral hygiene tools and techniques. The most traditional self‐care recommendation for interdental cleaning is using dental floss. However, the literature contains conflicting reports regarding flossing effectiveness. In 2015, it was agreed that the best evidence for effective interdental cleaning is available for IDBs. A meta‐review^5^ summarizing the available systematic reviews analysed the efficacy of interdental plaque removal devices in conjunction with normal toothbrushing (TB). Network meta‐analysis also indicated that interdental cleaning with IDBs was the most effective method for interdental plaque removal.^6^

If the IDB does not appropriately fit without trauma, room exists for other interdental cleaners. A relatively new interdental device is the rubber or elastomeric bristles interdental cleaner (RBIC). The first product was Soft‐pick®, marketed by the GUM® Company (Sunstar Europe S.A.). Its plastic core with soft elastomeric bristles was said to massage the gingiva and dislodge food. It is presented as an alternative to flossing and should improve patient compliance. A more recent development is a comparable product, EasyPick™, from the TePe® Company (Tepe Munhygienprodukter AB), where the core is firmly covered with a flexible silicone coating and lamellae.

The RBIC is different from a traditional toothpick (wood stick), which is commonly made from wood. The recently published Cochrane systematic review on interdental cleaning devices^7^ evaluates various rubber interdental cleaning devices but does not specifically evaluate the RBIC. This review includes rubber stimulators and an electronic powered interdental cleaning device, which is not even made of rubber.

The present systematic review evaluates the efficacy of the RBIC from publications available in the dental scientific literature to guide dental care professionals in evidence‐based decision making. The aim was to establish the efficacy of RBICs as an adjunct to TB compared to the adjuvant use of other interdental cleaning devices and TB alone based on dental plaque and gingival health parameters. Additionally, the safety aspects of the RBIC were evaluated as well as participants’ appreciation of the products used.

## 2 MATERIALS AND METHODS

This paper was prepared and reported in accordance with the Cochrane Handbook^8^ for Systematic Reviews of Interventions. Additionally, the guidelines of Transparent Reporting of Systematic Reviews and Meta‐analyses (PRISMA statement)^9^, ^10^ were used. The protocol was developed ‘a priori’ following an initial discussion between the research team members (PROSPERO # CRD42020172453).

### 2.1 Focused questions

Primary question:

- As an adjunct to TB, what is the efficacy of the RBIC compared to TB alone on dental plaque and gingival health parameters?

Secondary questions:

- As an adjunct to TB, what is the efficacy of the RBIC compared to other interdental cleaning devices on parameters of dental plaque and gingival health?
- Compared to other interdental cleaning devices, how safe is the RBIC?
- What is the panellists’ appreciation concerning the interdental cleaning devices evaluated?

### 2.2 Search strategy

Internet sources were used to search for appropriate papers that satisfied the study purpose. These sources included the National Library of Medicine, Washington, D.C. (MEDLINE‐PubMed), and the Cochrane Central Register of Controlled Trials (CENTRAL). A comprehensive search of the databases, using their query tools, was conducted through August 2020 for appropriate publications regarding the focused question. The terms included in the search strategy are presented in Table 1. Moreover, a manual search of the reference section of selected papers was performed.

**TABLE 1:** Search strategy. The asterisk (*) was used as a truncation symbol. The search strategy was customized according the database searched

Search terms used for PubMed‐MEDLINE: Intervention: {(rubber brush OR rubber cleaner OR rubber interdental* OR soft‐picks OR plastic brush OR plastic cleaner OR plastic interdental*) **AND** Outcome: (gingivitis OR periodontitis OR gingival pocket OR periodontal pocket OR gingival inflammation OR gingival diseases* OR periodontal diseases* OR bleeding on probing OR papillary bleeding index OR gingival bleeding OR bleeding index OR plaque removal OR plaque index OR dental plaque OR plaque OR removal OR interdental plaque OR interproximal plaque OR dental deposit* OR ‘Periodontal Diseases’ [MeSH terms])}

### 2.3 Screening and selection

Unique titles and abstracts of publications obtained from the searches were screened by two reviewers (NLHH and DES) using the Rayyan^11^ web application. The eligibility criteria were as follows:

- (Randomized) controlled clinical trials (CCTs or RCTs)
- Publications in English
- Publications conducted on humans
  ○ ≥18 years old
  ○ In good general health
  ○ Interdental cleaning performed by the participant
  ○ Without fixed orthodontic appliance
- Intervention: RBICs as an adjunct to TB
- Comparison: TB alone, IDBs, dental floss (DF) and other interdental cleaning devices
- Primary parameters of interest: plaque index (PI), bleeding score (BS) and gingival index (GI)
- Secondary parameters of interest: the safety of the interdental devices according to oral soft tissue (OST) assessments, adverse events (AEs) and gingival abrasion scores (GASs). Additionally, the participants’ appreciation concerning the products used was of interest.

During the screening process, the reviewers worked independently and were blinded to each other's results. Titles and abstracts were categorized as included, excluded or undecided. After the independent screening process, the search was unblinded, and the ‘conflicts’ identified by Rayyan^11^ were resolved by a discussion between the reviewers. Full‐text papers that fulfilled all the selection criteria were processed for data extraction. Attempts were made to contact the authors of the included publications to ask for additional data or information if these were unclear.

### 2.4 Assessment of heterogeneity

The heterogeneity of the primary outcome parameters across publications was detailed according to the following factors:

- Study design and participants’ characteristics
- Study procedures and products
- Indices and modifications

### 2.5 Quality assessment

Two reviewers (NLHH and DES) individually scored the methodological qualities of the included publications according to the method described in detail by Van der Weijden et al. (2009)^12^ and Keukenmeester et al. (2012).^13^ Disagreements regarding the screening and selection process were resolved by consensus or, if disagreement persisted, by arbitration through a third reviewer (GAW). Briefly, when random allocation, defined eligibility criteria, masking of examiners, masking of patients, balanced experimental groups, identical treatment between groups (except for the intervention), and reporting of follow‐up were present, the study was classified as having an estimated low risk of bias. When one of these criteria was missing, the study was considered to have an estimated moderate risk of bias. When two or more of these criteria were missing, the study was estimated to have a high risk of bias. The percentage of the items that met the quality standards was calculated. The estimated risk of bias was interpreted as follows: 0%–40% may represent a high risk of bias; 40%–60% may represent a substantial risk of bias; 60%–80% may represent a moderate risk of bias; 80%–100% may represent a low risk of bias.^5^ Separately, five ethical aspects were scored to explore whether the publications adhere to general ethical guidelines, such as funding and potential conflicts of interest.

### 2.6 Statistical analyses

#### 2.6.1 Data extraction

The data from the publications that met the selection criteria were extracted and processed for further analysis. Two reviewers (NLHH and DES) evaluated the selected publications for the mean baseline, end and incremental scores, and standard deviation (SD) or standard error (SE). If the SE was provided, the SD was calculated based on the sample size (SE = SD/√N). Disagreements were resolved by discussion, and if the disagreement persisted, the judgement of a third reviewer (GAW) was decisive. The original authors were contacted to ask for additional data.

#### 2.6.2 Data analysis

As a summary, a descriptive data presentation was used for all studies. The data were summarized and analysed using vote counting.^14^ The primary variables of interest were the PI, BS and GI. The secondary variables were safety and panellists’ appreciation of the evaluated products.

#### 2.6.3 Meta‐analysis

When appropriate and when including a minimum of two comparisons with the same intervention groups and design, a meta‐analysis was performed. The difference of means (DiffM) was calculated using an inverse variance method in Review Manager (RevMan)^15^ with either the fixed or random‐effects model, as appropriate. A *p‐value* of < 0.05 was considered significant. Heterogeneity was tested using the chi‐square test and the I^2^ statistic.^8^

### 2.7 Grading the ‘body of evidence’

The Grading of Recommendations, Assessment, Development and Evaluation (GRADE) was used to rank the evidence.^16^, ^17^, ^18^ Two reviewers (GAW and DES) rated the quality of the evidence and the strength and direction of the recommendations according to the following aspects: risk of bias, consistency of results, directness of evidence, precision, publication bias and magnitude of the effect. Any disagreement between the two reviewers was resolved through additional discussion.

## 3 RESULTS

### 3.1 Search and selection results

**FIGURE 1:**
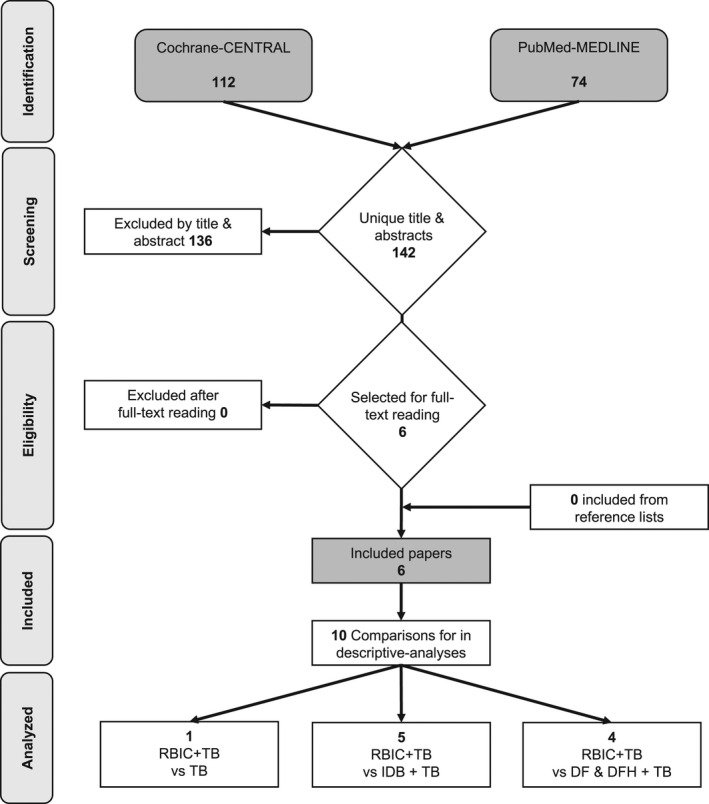
Search and selection results. DF, dental floss; DFH, dental floss holder; IDB, interdental brush; TB, toothbrush; RBIC, rubber bristle interdental cleaner

The searches provided six^19^, ^20^, ^21^, ^22^, ^23^, ^24^ publications, including 10 comparisons eligible for inclusion (for details, see Figure 1). The efficacy of the RBIC was compared to using an IDB (I, II, III, IV, VI), DF (I, IV, V) and DF in a holder (I) in conjunction with manual TB. So far, no comparisons to wood sticks have been published. Study IV involved a manual TB‐only group as a control.

### 3.2 Assessment of heterogeneity

Considerable heterogeneity was observed in the six clinical trials concerning the study design, evaluation period, study population, number, gender and age of participants. Information regarding the study characteristics is displayed in an overview in Table 2.

**TABLE 2:** Overview of the included studies and characteristics processed for data extraction

# Authors (year)	Study design, duration of experimental period Blinding Oral prophylaxis Funding	# Participants baseline (end) Gender Type of participants Mean age (SD) Age range in years	Groups Brand Dentifrice	Study procedures: Regimen use & instruction Professional/panellists brushing	Conclusions of the original authors
I Yost et al (2006)	RCT Parallel Examiner‐blind 6 weeks OP at baseline Single use Sunstar America	128 (120) ♀: 83 ♂: 37 (Sub)urban population Florida, USA 38.2 (?) 18–63	**RBIC:** GUM Soft‐Picks (Sunstar Americas Inc Chicago, USA) **IDB:** GUM Go‐betweens Cleaners (Sunstar Americas Inc Chicago, USA) **DF:** Crest Glide original (Procter & Gamble, Cincinnati, USA) **DFH:** Butler Flossers (Sunstar Americas Inc Chicago, USA) Used in all groups: **TB:** GUM 430 Soft (Sunstar Americas Inc Chicago, USA) **RDF:** Crest Regular (Procter & Gamble, Cincinnati, USA)	12–18 h non‐brushing before assessment Twice daily for 2 min Written manufacturers’ instructions Professional instruction Toothbrushing: frequency, duration and technique: NR Once daily interdental cleaning Brushing diary	No difference in plaque reduction and removal between DF, which is recognized as the golden standard, and DF, DFH, IDBs and RBICs.
II Abouassi et al (2014)	RCT Cross‐over Single‐blind 4 weeks OP at baseline Single use Funding: NR	51 (39) ♀: 16 ♂: 23 Recall patients from University of Freiburg, Germany 44 (?) 21–72	**RBIC:** Fuchs 3.5 mm with 0.9 mm PHD and 4.5 mm with 1.1 mm PHD (Interbros GmbH, Schönau Germany) **IDB:** parallel Red ISO 2, wire 0.5 mm PHD 0.9 mm and parallel Blue ISO 3, wire 0.6 mm PHD 1.1 mm (TePe, Malmö Sweden) Used in all groups: **TB:** Oral‐B indicator (Procter & Gamble, Weybridge, UK) **RDF:** Colgate Fresh Gel (Colgate‐Palmolive, Herstal, Belgium)	Overnight plaque before assessment Professional instruction Toothbrushing: frequency, duration and technique: NR Size RBIC/IDB tailored 4 weeks wash‐out	The RBICs showed more plaque accumulation compared to the IDBs, but with no statistical significance between the two devices. Both products showed a reduction in gingival inflammation after 4 weeks.
III Hennequin‐ Hoenderdos et al (2017)	RCT Parallel Examiner‐blind 4 weeks Split‐mouth OP at baseline Experimental‐gingivitis model Sunstar	44 (42) ♀: 31 ♂: 11 Young adults from Amsterdam, the Netherlands 23.2 (3) 19–33	**RBIC:** GUM Soft‐Picks Advanced (tip‐rear) Ø1.6 mm−3.7 mm (GUM Sunstar) **IDB:** parallel ISO 1 (tip‐rear) 2.0 mm−2.1 mm (GUM Sunstar) Used in all groups: **TB:** Butler GUM #311 Slender Soft (GUM Sunstar) **RDF:** HEMA (Amsterdam, the Netherlands)	2–3 h non‐brushing before assessment Professional instruction Twice daily 2 min brushing with the bass technique 2 weeks familiarization phase 21 days of non‐brushing the lower jaw Split mouth use IDB/RBIC in the lower jaw once daily Compliance diary	The RBIC, in conjunction with manual toothbrushing, was found to be more effective in reducing gingival inflammation after 4 weeks. The RBIC caused less abrasion of the gingiva and was appreciated more by the participants.
IV Graziani et al (2018)	RCT Parallel Examiner‐blind 4 weeks OP at baseline Italian Ministry Health and the Tuscan Region	60 (60) ♀: 29◊ ♂: 31 Young adults, from Pisa, Italy 26.9◊ (?) Range?	**RBIC:** GUM Soft‐Picks (Sunstar) **IDB:** size? (TePe, Malmö Sweden) **DF:** Dental Tape (TePe, Malmö Sweden) **TB:** TePe Select (TePe, Malmö Sweden) Used in all groups: **TB:** TePe Select (TePe, Malmö Sweden) **RDF:** ?	Plaque accumulation NR Professional instruction Brushing bass technique Frequency and duration: NR	Use of IDBs or RBICs reduces more interdental plaque in comparison with toothbrushing alone.
V Moretti et al (2020)	RCT Parallel Examiner‐blind 4 weeks OP at baseline Sunstar Americas, Incorporated	50 (49) ♀: 25 ♂: 25 Gingivitis patients from Chapel Hill, campus 26.6 ◊ (?) Range (18–70)	**RBIC:** GUM Soft‐Picks advanced (Sunstar Americas Inc Chicago, USA) **DF:** Oral‐B Pro Health Glide original (Procter & Gamble, Cincinnati, USA) **TB:** Oral‐B indicator manual brush (Procter & Gamble, Cincinnati, USA) **RDF:** Crest Coolmint gel dentifrice (Procter & Gamble, Cincinnati, USA)	12–18 h non‐brushing No chewing gum or crunchy food 3–6 h before assessment Appropriate written and professional instruction Toothbrushing: Twice daily brushing,once daily use interdental device Duration and technique: NR Brushing and experience diary	The RBIC was similar to Floss in clinical effectiveness. Ease of use of RBIC may have affected the participants’ motivation for interdental cleaning, resulting in better compliance.
VI Ustaoğlu et al (2020)	RCT Parallel Examiner‐blind 4 weeks Split‐mouth OP at baseline Funding: NR	34 (30) ♀: 18 ♂: 12 Gingivitis patients from Abant Izzet Baysal University 29.33 (2.21) Range (18–35)	**RBIC**♦: EasyPick™ XS/S and M/L (TePe, Malmö, Sweden) **IDB:** TePe® Interdental Brushes Original 9 wire sizes, ⌀ 0.4–1.5 mm (TePe, Malmö, Sweden) **TB:** Select™, (TePe ®, Malmö, Sweden) **RDF:** İpana Pro‐Expert®	Plaque accumulation NR Written instruction Toothbrushing: Twice daily brushing,and use interdental device Duration and technique: modified Stillman technique	The clinical efficiency of the tested interdental devices was similar in terms of removing plaque and decreasing bleeding. RBIC were found to be more comfortable and preferable to IDB.

◊: Calculated by the authors of this review based on the presented data in the selected paper;?: unknown; DF: Dental floss; DFH: Dental floss holder; RDF: Dentifrice; IDB: Interdental brush; ISO: International Organization for Standardization; TB: Manual toothbrush; NR: Not reported; OP: Oral prophylaxis; PHD: Passage hole diameter; RBIC: Rubber bristle interdental cleaner; RCT: Randomized controlled trial, ♦= plastic core with flexible silicone coating and lamellae.

#### 3.2.1 Study design and participants’ characteristics

Of the six selected RCTs, five had a parallel (I, III, IV) and one a crossover (II) design. One RCT (III) used the reversal of an experimental gingivitis model. The duration varied from 4 to 6 weeks. Two studies also assessed the plaque level before and after using the interdental devices (single‐use effect). Two studies used a split‐mouth design (III, VI), whereby the third quadrant (left side) and the fourth quadrant (right side) were cleaned interdentally as randomly assigned. The total number of participants analysed ranged from 39 to 120. The approximate mean age of the 340 participants was 33 and varied from 18 to 72. The participants were in good general health and had gingivitis (II, V, VI) but no periodontitis, as defined in the inclusion and exclusion criteria (I, III, IV). Five of the included studies (I, II, IV, V, VI) reported information regarding the participants’ interdental cleaning dexterity and experience. In Study I, the participants could floss but were not current floss‐users. Study II required participants with mechanical skills to use interdental devices. Eligible participants in Study III did not use an interdental device as part of their daily oral hygiene procedure. Study IV did not report information concerning dexterity; however, participants with intact interdental papillae were selected. Five included studies selected participants for which the interdental device fitted. Study I required ≥5 evaluable interdental sites; Study III required ≥8 interdental spaces, four per quadrant; Study V required 12 interdental spaces; Study II required ≥18 interdental sites, and Study VI evaluated interdental spaces from the incisors to the second molars.

#### 3.2.2 Study procedures and products

All studies provided oral prophylaxis at baseline. The reported period of non‐brushing before measurements varied from 2–3 (III) to 12–18 (I, V) hours. In three studies, it was unclear how many hours participants performed (interdental) cleaning before assessment (II, IV, VI). Four studies evaluated RBICs from Soft‐pick® (I, III, IV, V), marketed by the GUM® Company, and one study evaluated those marketed by the Fuchs® Company (II). One study evaluated interdental cleaners with flexible silicone coating and lamellae (Study VI, EasyPick™ from the TePe® Company). In all studies, participants were instructed to use a manual toothbrush and a dentifrice in addition to their interdental cleaning device. Detailed information concerning the products is presented in Table 1. Three of the included studies (II, III and V) reported information regarding the participants’ interdental cleaning dexterity and experience but provided no sub‐analysis on this aspect.

#### 3.2.3 Indices and modifications

The Turesky et al. (1970)^25^ Modified Quigley and Hein (1962)^26^ (TMQH) **PI** was used in four experiments (II, III, V, VI). This index has a scale of 0–5 and was originally scored at two (buccal and lingual) tooth sites, as in Study II. Study III scored six surfaces per tooth according to the modification by Lobene (1982),^27^ and Study V scored only interdental spaces. The TMQH modification by Benson et al. (1993)^28^ was used in Study I, in which the segments were categorized as distal proximal, marginal and mesial proximal. In Study IV, the Plaque Control Record of O’ Leary et al. (1972)^29^ was used; however, it was scored at six surfaces rather than the original four surfaces per tooth.

Two studies (I, V) used the **GI** of Löe and Silness (1963)^30^ on a scale from 0 to 3. For the **BS**, two studies assessed the Eastman Interdental Bleeding Index of Caton and Polson (1985)^31^ (I & II). The Bleeding On Marginal Probing (BOMP) Index, also called the Angular Bleeding Index (AGI), was assessed in two studies (III, IV), according to Van der Weijden et al. (1994).^32^ The Papillary Bleeding Index (PBI) of Saxer and Mühlemann (1975)^33^ was assessed by Study VI, and Study IV also recorded the absence and presence of gingival bleeding according to Ainamo and Bay (1975).^34^

Concerning safety assessments, the **GAS** was assessed in one study, according to Van der Weijden et al. (2004).^35^, ^36^, ^37^, ^38^ Study I performed an **OST assessment** at baseline and after 6 weeks. Further details were missing. Study III reported all AEs, which were followed until they had abated or a stable situation was reached. Study II evaluated the acute pain intensity when using the product on a 10‐point Likert scale. To assess panellists’ appreciation of using the study products, a questionnaire with a **visual analog scale** (VAS)^13^ was completed to assess patients’ perception after completing Study III. In Studies II and VI, participants were questioned afterwards regarding their acceptance and satisfaction using the product on a 5‐point Likert scale.

### 3.3 Quality assessment

To estimate the risk of bias in the included publications, the quality assessment included internal validity, external validity, statistical validity, and clinical and ethical aspects, as presented in online Appendix S1. The estimated potential risk of bias was low for all publications. It was shown that the five ethical aspects were more frequently reported in the publications from the last decade compared to Study I from 2006. Three studies were funded by SUNSTAR America (I, III, V) and one study by the Italian Ministry of Health and the Tuscan region (IV). The funding of two studies is unknown (II, VI). All authors were affiliated academics. In four studies (II, III, IV, VI), the authors explicitly stated that they had no conflicts of interest.

### 3.4 Results of study outcomes

Online Appendix S2 shows the results from the data extraction of PI, BS and GI scores of the selected publications. Table 3A presents a descriptive summary comparison and intervention indicating the significances of the primary parameters. Generally, no statistically significant differences were found. Table 3B summarizes the secondary parameters.

**TABLE 3:** Descriptive summary of the comparison and intervention indicating significances of the (A) primary parameters as found in the original papers and (B) secondary parameters as found in the original papers

(A)										
# Authors (year)	TB+ Intervention	PI			BS		BOMP		GI	TB+ Comparison
		Single‐use comparisons	Follow‐up comparisons							
IV Graziani et al (2018)	RBIC		O	+*	O	O*	O	O*		TB only
I Yost et al (2006)	RBIC	O	O		O				O	DF
I Yost et al (2006)	RBIC	O	O		O				O	DFH
IV Graziani et al (2018)	RBIC		O	O*	O	+*	O	O*		DF
V Moretti et al. (2020)	RBIC		O*		O				O*	DF
I Yost et al (2006)	RBIC	O	O		O				‐	IDB
II Abouassi et al (2014)	RBIC	‐ *	O*		O*					IDB
III Hennequin‐Hoenderdos et al (2017)	RBIC		O	O*			O	+*		IDB
IV Graziani et al (2018)	RBIC		O	O*	O	O*	O	O*		IDB
VI Ustaoğlu et al (2020)	RBIC		O		O					IDB

(B)							
# Authors (year)	TB+ intervention	Safety				Panellist Preference/Ease of use	TB+ Comparison
		OST	AE	GA	Pain		
I Yost et al (2006)	RBIC	?					DF
I Yost et al (2006)	RBIC	?					DFH
V Moretti et al (2020)	RBIC	O	O			+	DF
I Yost et al (2006)	RBIC	?					IDB
II Abouassi et al (2014)	RBIC				+	+	IDB
III Hennequin‐Hoenderdos et al (2017)	RBIC		NRttSP	+		+	IDB
VI Ustaoğlu et al (2020)	RBIC				+	+	IDB

Abbreviations: AE, adverse events; BOMP, bleeding on marginal probing; BS, bleeding score; DF, dental floss; DFH, dental floss holder; GA, Gingival Abrasion score; GI, gingival index; IDB, interdental brush; OST, Oral Soft Tissue examination; PI, plaque index; RBIC, rubber bristle interdental cleaner; TB, toothbrush.

* Analysing interdental/accessible sites.

#### 3.4.1 Primary parameters

Most studies indicated no difference between using DF, IDBs or RBICs in plaque scores. One single‐use study found the IDB to be more effective than the RBIC (Study II). This was not the case in the five comparisons with a long follow‐up (I, II, III, IV, VI), where no differences between RBICs and IDBs were found for plaque removal. In one study with a follow‐up design, the adjuvant use of the RBIC compared to TB resulted in a significantly lower plaque score in sites accessible with the RBIC (Study IV). Most studies indicated no difference between using DF, an IDB, or an RBIC in the GI or BS. In two studies, the adjuvant use of the RBIC resulted in a significantly lower interdental BS for the RBIC group compared to DF (Study IV) and the IDB (Study III). However, no significant difference was observed if whole mouth scores were compared. In one study, which evaluated gingival health according to the GI, the IDB was more effective than the RBIC.

#### 3.4.2 Meta‐analysis

**FIGURE 2:**
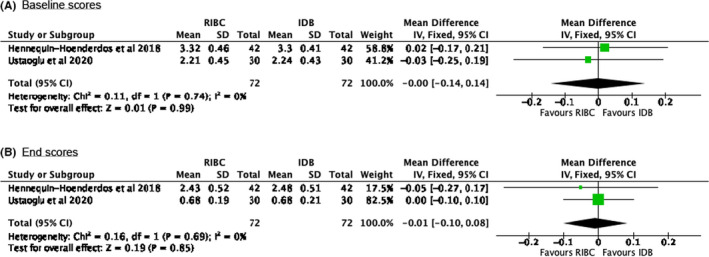
Forrest plots of the meta‐analysis for the Tureskey (1970) modification of the Quigley & Hein (1962) plaque score measured. Presented for the baseline and end scores, using a fixed effects model. A chi‐square test resulting in a *p*‐value < 0.1 was considered to be an indication of significant statistical heterogeneity. As an approximate guide for assessing the degree of inconsistency across studies, an I^2^ statistic of 0%–40% was interpreted as might not be important, a statistic of 40%–60%% as possibly representing moderate heterogeneity, 60%–80% as possibly representing substantial heterogeneity and 80%–100% as possibly representing considerable heterogeneity.

**FIGURE 3:**
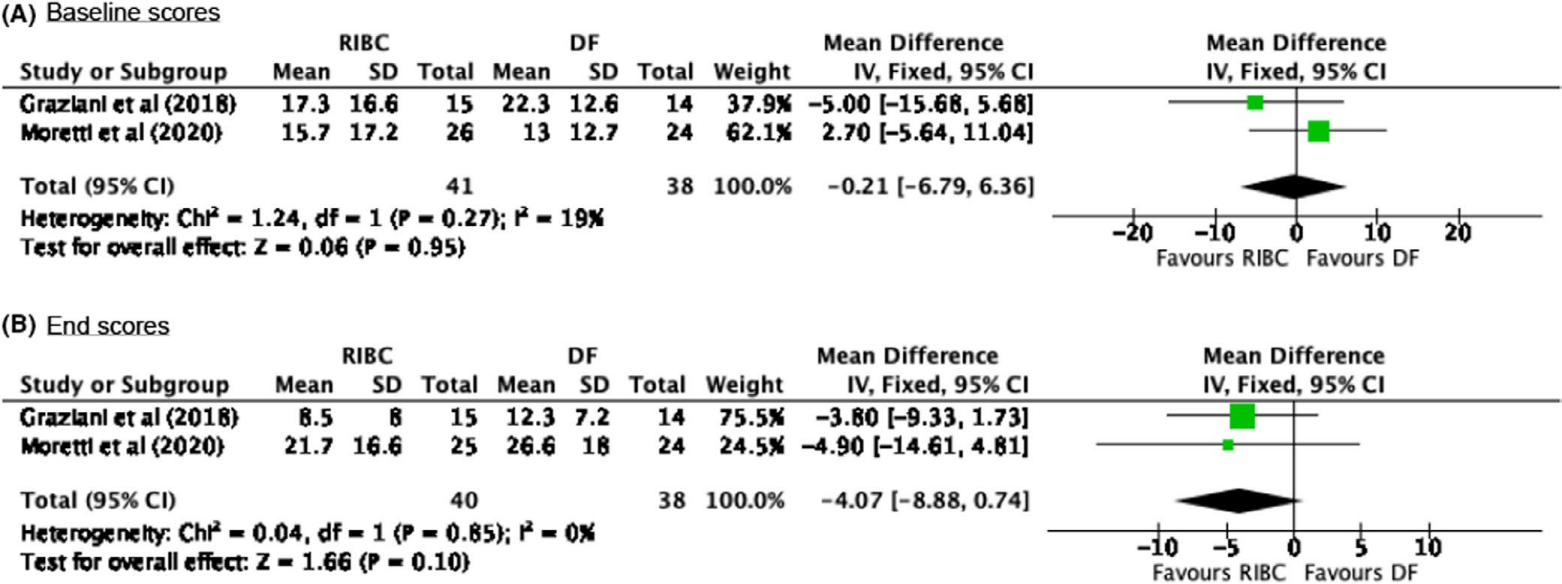
Forrest plots of the meta‐analysis for the percentage bleedings score measured. Presented for the baseline and end scores, using a fixed effects model. A chi‐square test resulting in a *p*‐value < 0.1 was considered to be an indication of significant statistical heterogeneity. As an approximate guide for assessing the degree of inconsistency across studies, an I^2^ statistic of 0%–40% was interpreted as might not be important, a statistic of 40%–60%% as possibly representing moderate heterogeneity, 60%–80% as possibly representing substantial heterogeneity and 80%–100% as possibly representing considerable heterogeneity

Figures 2 and 3 present forest plots of the outcomes of the meta‐analysis, including two comparisons of plaque removal scores (III, VI), in which the RBIC is compared to the IDB, and two of BS (IV, V) evaluating the RBIC and DF. For the comparison of plaque scores, no significant difference between the RBIC and IDB was found (−0.01, 95% CI: [−0.10; 0.08]). Regarding BSs, the comparison between the RIBC and DF showed no significant difference between these two interdental oral hygiene products (−4.07, 95% CI: [−8.88; 0.74]). For other clinical parameters, meta‐analysis was infeasible due to a lack of adequate and useable data from the included studies. Regarding the potential publication bias, the meta‐analysis comprised an insufficient number of trials to enable meaningful visual inspection of the funnel plot.

#### 3.4.3 Secondary parameters

Study III reported four adverse events related to the clinical trial, which concerned (brushing) trauma of the gingiva. Assessment of gingival abrasions on the oral soft tissue resulted in a statistically significant difference between the RBIC and the IDB group. Fewer abrasions were reported in the RBIC group (online Appendix S2E). In Study V, no adverse events were reported during the trial. Two studies (I, IV) reported no side effects. Studies II and VI assessed the level of pain when using an IDB compared to an RBIC. The RBIC was rated as significantly more comfortable and softer than the IDB. It achieved significantly higher scores regarding panellists’ preference in overall assessment and subitems for comfort and ease of brushing (II, III, V, VI). For details, see online Appendix S2F.

### 3.5 Evidence profile

Table 4 presents a summary of the various outcomes with which the quality of the evidence was rated and with which the strength and direction of recommendations were appraised. Given the strength of the recommendation, a weak to very weak certainty exists that the RBIC is indicated for gingivitis and plaque reduction. The evidence supports user safety and participants’ preference.

**TABLE 4:** Summary of Findings table based on the quality and body of evidence on the estimated evidence profile and appraisal of the strength of the recommendation regarding the efficacy of RBIC on the parameters of interest

Study design	Plaque	Bleeding	Gingivitis	Safety	Preference
	RCT	RCT	RCT	RCT	RCT
# Experiments descriptives analysis (Table 3)	4 Single use 10 Follow‐up	10	9	6	4
Risk of bias (online Appendix S1)	Low	Low	Low	Low	Low
Consistency	Rather consistent	Rather consistent	Rather consistent	Rather consistent	Consistent
Directness	Direct	Direct	Direct	Direct	Direct
Precision	Imprecise	Imprecise	Imprecise	Imprecise	Rather imprecise
Reporting bias	Possible	Possible	Possible	Possible	Possible
Magnitude of the effect (Table 3)	No difference	No difference	No difference	No difference	In favour of RBIC
Strength and direction of the recommendation	Weak quality evidence for no difference	Weak quality evidence for no difference	Very weak quality evidence for no difference	Very weak quality evidence for no difference	Very weak quality evidence in favour of
**Overall recommendation**	If an interdental device is indicated for gingivitis and plaque reduction, there is weak to very weak evidence for RBICs to recommend as a product. RBIC is considered to be safe and well accepted.				

## 4 DISCUSSION

Removing bacterial plaque is considered a key approach to preventing and controlling periodontal diseases. Toothbrushing generally cannot clean interproximal sites effectively. Therefore, interdental cleaning aids play a vital role in optimizing gingival health and preventing oral disease. Various interdental aids are available. Rubber bristles interdental cleaners are a relatively recent development and could be a viable alternative to other interdental cleaning devices. This systematic review aimed to establish, based on the existing literature, the clinical efficacy of the RBIC regarding dental plaque and gingival health parameters, as well as safety aspects and participants’ appreciation. Only a few clinical trials have evaluated this new device. The six eligible studies retrieved only allowed a descriptive data analysis of most of the parameters due to heterogeneity in design and variations in the indices used as outcome parameters. Only meta‐analysis that included two experiments was feasible, one for plaque scores and the other regarding BSs. Synthesis of the available evidence showed that in gingivitis patients, a minimal difference existed between using an RBIC, IDB or DF in conjunction with TB in clinical parameters of plaque and gingivitis. Compared to the IDB, the RBIC led to fewer gingival abrasions and was appreciated more by participants. However, because the duration of the included studies was short, longer‐term studies are needed to enable conclusions regarding oral health benefits.

### 4.1 Patient appreciation

By disturbing and removing plaque, flossing has become the standard of interdental care. Dental floss reaches the area where gingivitis starts and which is often missed when brushing. However, floss is difficult to use. This presents a barrier to achieving good oral care to those with reduced dexterity or inability or unwillingness to devote time to flossing.^21^ In earlier work,^39^ we found that the participants’ preference for IDBs was higher than for floss. The IDB was considered more efficacious. This higher level of efficacy was confirmed in a systematic review^40^ indicating that most studies found a significant positive difference in the plaque score reduction, favouring the IDB over floss. Regarding participants’ appreciation, the four included studies that assessed this aspect (II, III, V, VI) found that the participants preferred the RBIC over the IDB. The RBIC also received higher scores for comfort and ease of brushing (II, V, VI) and willingness to buy the product (I, VII). The RIBC also showed high patient compliance, probably due to its soft internal structure (II). Satisfaction with the RBIC is not unexpected, as many individuals seek products that are quick and easy to use. A recent study assessed the knowledge, attitudes and behaviours of patients regarding interdental cleaning devices. Participants reported challenges with the RBIC, such as pain and irritation during use and difficulty cleaning between teeth effectively; the end was too thick, or the length was too short to reach the back teeth. Some disliked the taste or that it was single‐use only.^41^ Patient acceptance is an important issue regarding long‐term adherence to using interdental cleaning devices.^42^

### 4.2 Interdental plaque

Direct investigation of the plaque removal performance ‘*in vivo’* is impossible because interproximal spaces in a closed dental arch are not directly visible.^43^ Hence, researchers must rely on ‘*in‐vitro*’ models that allow removal of the teeth and visualization of the facing interproximal surfaces. An ‘*in‐vitro*’ study evaluated 72 extracted human teeth, grouped as incisors, premolars and molars, and embedded in acrylic resin. To imitate the interdental plaque, the interproximal areas of the teeth were dyed with contact spray. After applying interdental devices, the interproximal surfaces of the teeth were digitally photographed, and dye removal was calculated. This study revealed that the plaque removal efficacy of the IDB was better than that of the RBIC (Tepe EasyPick^TM^ Malmo, Sweden) and wood stick.^44^ It was proposed that IDBs are effective for the central part of the interdental space, as bristles of an appropriately sized brush can disrupt the interdental oral biofilm, especially in the concave tooth and root anatomy of premolars and molars.^40^ Adequate ‘*in‐vivo*’ models are needed to study this supposition in more detail and allow generalizable evaluation of different interdental cleaning devices.

### 4.3 Periodontitis

The most appropriate interdental hygiene aids must be selected for each patient, with the choice depending mainly on the size and shape of the interdental spaces and the morphology of the interdental surfaces.^45^ As an RBIC visually resembles an IDB, the indication for its use may easily be mistaken. The RBIC is more like a toothpick than an IDB. The small elastomeric fingers or lamellae probably do not contribute to efficacy in plaque removal, and based on clinical experience, they can easily become detached when the RBIC is used. Consequently, RBICs are designed to mechanically remove the plaque from interdental surfaces through the friction of the sides rubbing against the interproximal tooth surfaces. It is noteworthy that the participants in four of the included studies (III, IV, V, VI) were young and probably had interdental spaces filled with an interdental papilla. In a study by Hennequin‐Hoenderdos et al. (2017),^20^ only one size of IDB and RBIC was studied. However, Ustaoğlu et al. (2020)^22^ studied two sizes of RBIC and nine sizes of IDB. However, both studies reached the same conclusion that no difference existed between RBICs and IDBs in young gingivitis patients. It would be interesting to evaluate both RBICs and IDBs in periodontitis patients with wide interdental spaces. Hypothetically, IDBs may be advantageous because they are available for the smallest to the largest interdental spaces with brush diameters from 1.9 to 14 mm. An IDB that is sized correctly for each interdental space is easy to handle and will be atraumatic to the papillae.^5^ Based on the outcome of the present review, we propose the RBIC as an alternative for an IDB in gingivitis patients. For periodontitis patients, no evidence currently exists. A recent systematic review concluded that, due to the scarcity of studies that met the inclusion criteria for each of the oral hygiene devices and the low certainty of the resultant evidence, no strong ‘evidence‐based’ conclusion could be drawn concerning any specific oral hygiene device for patient self‐care in periodontal maintenance.^46^

### 4.4 Accessibility

Although in young and healthy people, most interdental sites can be cleaned using IDBs,^47^ two studies (III, IV) evaluated accessible sites in which the assigned products could be inserted. In these sub‐analyses, one paper described a significant difference regarding reducing gingival inflammation in favour of the RBIC compared to floss (Study IV), while the results of another favoured the IDB (Study III). However, full mouth analysis in both studies (III, IV) showed no difference between the RBIC and the IDB.

### 4.5 Ethical consideration

The ethical aspects of biomedical research have received increasing interest. It is recognized that appropriate consideration of clinical research ethics is closely associated with the methodological quality of clinical trials.^48^ The results of clinical trials conducted under conditions incompatible with proper respect for the person participating as a panellist in research would be unacceptable to circulate and use. Paradoxically, the ethical aspects of systematic reviews are seldom assessed. This can probably be explained by their secondary nature. It is erroneous to presume that the original studies included in systematic reviews automatically respect the fundamental ethical criteria. A comprehensive search for available evidence for systematic reviews does not prevent the inclusion of unethical studies.^49^ Including ethical aspects as an integral part of the quality assessment is under debate. The Cochrane handbook^8^ states that measures of ‘quality’ alone are often strongly associated with aspects that could introduce bias. It is stated that criteria related to ethical aspects should not be assessed within this domain. However, including ethical aspects in the systematic review increases awareness of the ethical standards upheld in the underlying evidence. At the level of a systematic review, assessment of ethical issues should consider the time and place of the study. Older trials may have been conducted with appropriate ethical standards of research but are not as clearly reported as they are today. Insufficient reporting of ethical issues in original papers does not necessarily mean that the studies were not conducted based on ethical principles. Therefore, Weingarten et al. (2004)^50^ state that reviews should at least include a report on the ethical assessment. Further research should address the ethical gaps observed in existing studies. Clinical trials are undertaken to benefit patients and, when properly performed, add to the evidence available in the public domain. However, outcomes are sometimes not reported because trials may have stopped due to negative or equivocal results. This may introduce a publication bias. In this respect, registration in a clinical trial registry is an ethical issue to address because only three (II, II, V) out of six papers mention this.

### 4.6 Hawthorne and novelty effect

A limitation that cannot be overcome is that the participants in the included studies could not be blinded regarding the devices used. This may have affected the participants’ behaviour regarding the novel device. Abouassi et al. (2014)^19^ studied plaque levels after a single brushing exercise at the start of the study; these were significantly different between the RBIC and IDB groups in favour of the IDB, which was not substantiated in the follow‐up assessment. This is probably accountable to an element of the ‘Hawthorne effect’ or ‘novelty effect’ of using a new device, which heightens the motivation to use a product.^51^ This effect probably weakened over the study period because, after 4 weeks of home use, no differences between the groups were found regarding the plaque and gingival parameters.

### 4.7 Limitation

Several limitations can be identified for this review. Interpretation of the research literature was limited by factors including short duration, industry involvement, heterogeneity of study designs, brands used and assessment parameters. Moreover, the groups of participants in this review comprised healthy individuals with low levels of disease and low levels of inflammation at baseline. Therefore, the findings are not necessarily generalizable to patients exhibiting high levels of inflammation. Although English is commonly accepted as the language of scientific research, relevant external evidence could also arise from studies in languages other than English.^52^ The influence of language restrictions on the outcome of systematic reviews is uncertain.^52^ However, the exclusion of non‐English‐language studies from our systematic review may have led to a language bias.^53^

In comparison with IDBs, three different RBIC product types were used. These mainly differed according to their surface texture, for which the impact of the plaque removal ability is currently not revealed. The results of the meta‐analysis should therefore be interpreted with caution, as this is a synthesis of two comparisons with two different products.

## 5 CONCLUSION

Based on a descriptive and a meta‐analysis of the available literature, it is synthesized that in gingivitis patients, a weak to very weak certainty exists that a RBIC is indicated for gingivitis and plaque reduction. The evidence supports user safety and participants’ preferences.

## 6 CLINICAL RELEVANCE

### 6.1 Scientific rationale for the study

The RBIC is a relatively new interdental cleaning device. For an evidence‐based recommendation, an overview of the available scientific literature is required.

### 6.2 Principal findings

In conjunction with toothbrushing, minimal differences exist between using RBICs, IDBs and DF on plaque and gingivitis in gingivitis patients. Participants expressed their preference for RBICs.

### 6.3 Practical implications

Overall, the evidence suggests that RBICs may be recommended as alternative interdental cleaning devices for gingivitis patients. Because the duration of the included studies was short, longer‐term studies are needed to allow conclusions on oral health benefits.

## CONFLICT OF INTEREST

The authors declare that they have no conflicts of interest. The authors have previously received either external advisor fees, lecturer fees or (unrestricted) research grants from oral hygiene products manufacturers. Those manufacturers included Colgate, Curaprox, Dentaid, GABA, Johnson & Johnson, Lactona, Oral‐B, Philips, Procter & Gamble, Sara Lee, Sunstar, TePe, Unilever and Waterpik.

## AUTHOR CONTRIBUTION

GAW contributed to conception and design, analysis and interpretation, drafted and critically revised the manuscript. DES contributed to conception and design, search and selection, analysis and interpretation, and critically revised the manuscript. EVDS was involved in analysis and interpretation and critically revised the manuscript. NLHH contributed to design, search and selection, analysis and interpretation, and drafted the manuscript. All authors gave final approval and agreed to be accountable for all aspects of work ensuring integrity and accuracy.

## Supporting information

Appendix S1‐4

## Data Availability

This is a systematic review, data were already published in the paper that are included.
