# Supplementary material for: The efficacy of a rubber bristles interdental cleaner on parameters of oral soft tissue health‐a systematic review‐
Source: Int J Dent Hyg. 2021 May 29;20(1):26–39. doi: 10.1111/idh.12492 (PMC9292806; doi:10.1111/idh.12492)
Supplement: Supplementary file 1 — Appendix S1‐4 [file IDH-20-26-s001.docx]

To be published in: International Journal of Dental Hygiene

Version: 02 September-2020

**APPENDICES**

### The efficacy of rubber bristle interdental

### cleaners on parameters of the gingival condition

***a systematic review***

**Online supporting information legends**

*Additional supporting information can be found in the online version of this article.*

**Appendix S1.** Methodological, validity and quality scores and estimated risk of bias of the included studies.

**Appendix S2-A.** Overview of **Plaque Index scores after 4/6 weeks use** of the selected studies with various indices and their modifications. Baseline, end measurements and differences are presented as the (adjusted) means and standard deviations (SD) shown in parentheses. Statistically significant changes within groups are presented.

**Appendix S2-B.** Overview of **Plaque Index scores after single use** of the selected studies with various indices and their modifications. Baseline, end measurements and differences are presented as the (adjusted) means and standard deviations (SD) shown in parentheses. Statistically significant changes within groups are presented.

**Appendix S2-C.** Overview of **Bleeding Scores** of the selected studies with various indices and their modifications. Baseline, end measurements and differences are presented as the (adjusted) means and standard deviations (SD) shown in parentheses. Statistically significant changes within groups are presented.

**Appendix S2-D.** Overview of **Gingival Index scores** of the selected studies with various indices and their modifications. Baseline, end measurements and differences are presented as the (adjusted) means and standard deviations (SD) shown in parentheses. Statistically significant changes within groups are presented.

**Appendix S2-E.** Overview of **Gingival Abrasion Scores** of the selected studies with various indices and their modifications. Baseline, end measurements and differences are presented as the (adjusted) means and standard deviations (SD) shown in parentheses. Statistically significant changes within groups are presented.

**Appendix S2-F.** Overview of **Panelist Preference assessment** of the selected studies.

**Appendix S3.** PRISMA checklist.

**Appendix S4.** References online appendixes.

**Appendix S1.** Methodological, validity and quality scores of the included studies.

| **Study**  **Quality criteria** | | | **I**  **Yost et al**  **(2006)** | **II**  **Abouassi et al**  **(2014)** | **III**  **Hennequin-Hoenderdos**  **et al (2017)** | **IV**  **Graziani et al**  **(2018)** | **V**  **Moretti et al. (2020)** | **VI**  **Ustaoğlu et al (2020)** |
| --- | --- | --- | --- | --- | --- | --- | --- | --- |
|  | Study design | | Parallel | Crossover | Parallel | Parallel | Parallel | Parallel |
| **Internal validity** | Random allocation * | | + | + | + | + | + | + |
|  | Allocation concealment | | - | - | + | + | - | - |
|  | Blinded to product * | | NA | NA | NA | NA | NA | NA |
|  | Blinded to examiner * | | + | + | + | + | + | + |
|  | Blinding during statistical analysis | | - | - | + | - | - | - |
|  | Balanced experimental groups * | | + | + | + | + | + | + |
|  | Reported loss to follow up* | | + | + | + | + | + | + |
|  | # (%) of drop-outs | | N=8 (6.3%◊) | N=12 (23.5%◊) | N=0 (0%◊) | N=0 (0%◊) | N=1 (2%◊) | N=0 (0%◊) |
|  | Treatment identical, except for intervention * | | + | + | + | + | + | + |
| **External validity** | Representative population group | | + | + | + | + | + | + |
|  | Eligibility criteria defined * | | + | + | + | + | + | + |
|  | Sample size calculation and power | | - | + | + | + | + | + |
|  | Point estimates presented for the primary outcome | | + | + | + | + | + | + |
|  | Measures of variability presented for the primary outcome | | - | - | + | + | + | + |
|  | Unit of analysis | | ? | subject | subject | subject | subject | subject |
|  | Include an per protocol analysis | | + | + | + | + | - | + |
|  | Include an intention- to-treat analysis | | - | - | + | - | + | - |
|  | Correction for multiple comparisons | | ? | ? | ? | ? | ? | ? |
| **-ADA interdental**  **Cleaning guideline** | | Study duration > 30 days | + | + | + | + | + | + |
|  |  | Control (additional use of the manual interdental cleaner) group | - | - | - | + | - | - |
|  |  | Safety assessments | + | - | + | + | + | - |
|  |  | Plaque assessments | + | + | + | + | + | + |
|  |  | Gingivitis assessments | + | + | + | + | + | + |
| **Clinical aspects** | | Validated measurement | + | + | + | - | + | + |
|  |  | Calibration examiner | - | + | + | - | + | + |
|  |  | Reproducibility data shown | - | - | - | - | - | + |
| **Ethical aspects** | | Approval of Medical Ethical Committee | ? | + | + | + | + | + |
|  |  | Registration in Clinical Trail Registry | - | + | + | - | + | - |
|  |  | Informed consent | + | + | + | + | + | + |
|  |  | Conflict of Interest reported | - | + | + | + |  | + |
|  |  | Author(s) from the industry involved | + | - | - | - | - | - |
|  |  | Funding reported | + | - | + | + | + | - |
| **Authors estimated risk of bias** | | | low | low | low | low | low | low |

Each aspect of the score list was given a rating of ‘+’ for an informative description of the item concerned and a study design meeting the quality standard, ‘-’ for an informative description without a study design that met the quality standard, and ‘?’ for lacking or insufficient information. When random allocation, defined eligibility criteria, blinding of examiners and patients, balanced experimental groups, identical treatment between groups (except for intervention), and report of follow-up were present, the study was classified as having a low risk of bias. When one of these seven criteria was missing, the study was considered to have a moderate potential risk of bias. When two or more of these criteria were missing, the study was considered to have a high potential risk of bias, as proposed by Van der Weijden et al. (2009).

? = not specified/unclear; + = yes; - = no; * = reporting criteria for estimation the potential risk of bias, NA=not applicable

**Appendix S2-A.** Overview of **Plaque Index scores after 4/6 weeks follow up** of the selected studies with various indices and their modifications. Baseline, end measurements and differences are presented as the (adjusted) means and standard deviations (SD) shown in parentheses. Statistically significant changes within groups are presented.

| **#**  **Authors (year)** | **Indices** | **Surfaces** | **Groups** | **Mean (SD)** | | | **Significant**  **within groups** |
| --- | --- | --- | --- | --- | --- | --- | --- |
|  |  |  |  | **Baseline** | **End** | **Difference** |  |
| **I**  **Yost**  **et al (2006)** | Benson modification (1993) of the Quigley & Hein (1962) Plaque Index | All | RBIC  IDB | 2.34 (?)  2.30 (?) | 1.95 (?)  1.84 (?) | -0.39◊ (?)  -0.46◊ (?) | yes  yes |
|  |  |  | RBIC  DF | 2.34 (?)  2.46 (?) | 1.95 (?)  2.06 (?) | -0.39◊ (?)  -0.40◊ (?) | yes  yes |
|  |  |  | RBIC  DFH | 2.34 (?)  2.60 (?) | 1.95 (?)  1.98 (?) | -0.39◊ (?)  -0.62◊ (?) | yes  yes |
| **II**  **Abouassi**  **et al (2014)** | Turesky (1970) modification of the Quigley & Hein (1962) Plaque Index | Accessible | RBIC  IDB | 1.88 (0.40♦)  1.91 (0.37♦) | 2.02 (0.42♦)  2.00 (0.42♦) | +0.13♦ (0.06♦)  +0.09♦ (0.06♦) | yes  no |
| **III**  **Hennequin-Hoenderdos**  **et al (2017)** | Turesky (1970) modification  of the Quigley and Hein plaque index (1962) according Lobene (1982) | All | RBIC  IDB | 3.32 (0.46)  3.30 (0.41) | 2.43 (0.52)  2.48 (0.51) | -0.89◊ (?)  -0.82◊ (?) | ?  ? |
|  |  | Accessible  mesial & distal | RBIC  IDB | 3.34 (0.55)  3.32 (0.46) | 2.55 (0.64)  2.57 (0.54) | -0.79◊ (?)  -0.75◊ (?) | ?  ? |
| **VI**  **Ustaoğlu**  **et al (2020)** | Turesky (1970) modification  of the Quigley and Hein plaque index (1962) | All | RBIC  IDB | 2.21 (0.45)  2.24 (0.43) | 0.68 (0.19)  0.68 (0.21) | -1.53◊ (?)  -1.56◊ (?) | Yes  yes |
| **V**  **Moretti**  **et al (2020)** | Turesky (1970) modification  of the Quigley and Hein plaque index (1962) according Lobene (1982) | Accessible | RBIC  DF | 3.35 (1.43)  3.65 (1.15) | 3.39 (1.31)  3.88 (0.87) | +0.04 (1.19)  +0.23 (0.93) | no  no |
| **IV**  **Graziani**  **et al (2018)** | Plaque Control Record  O' Leary (1972) | All | RBIC  TB | 22.1% (14.9%)  39.7% (19.5%) | 11.3% (8.0%)  21.5% (16.1%) | -10.8%◊ (?)  -18.2%◊ (?) | yes  yes |
|  |  |  | RBIC  IDB | 22.1% (14.9%)  18.9% (16.7%) | 11.3% (8.0%)  12.3% (11.1%) | -10.8%◊ (?)  -6.6%◊ (?) | yes  no |
|  |  |  | RBIC  DF | 22.1% (14.9%)  39.5% (20.0%) | 11.3% (8.0%)  20.6% (10.2%) | -10.8%◊ (?)  -18.9%◊ (?) | yes  yes |
|  |  | Interdental | RBIC  TB | 43.2% (32.0%)  54.2% (28.0%) | 19.7% (13.3%)  41.5% (35.0%) | -23.5%◊ (?)  -12.7%◊ (?) | no  no |
|  |  |  | RBIC  IDB | 43.2% (32.0%)  31.7% (17.2%) | 19.7% (13.3%)  19.1% (10.5%) | -23.5%◊ (?)  -12.6%◊ (?) | no  no |
|  |  |  | RBIC  DF | 43.2% (32.0%)  64.2% (33.4%) | 19.7% (13.3%)  29.9% (12.3%) | -23.5%◊ (?)  -34.3%◊ (?) | no  no |

?: unknown; ◊: calculated by the authors of this review based on the presented data in the selected paper; ♦: obtained by the original authors; SD: standard deviation

RBIC: Rubber bristle interdental cleaner; IDB: Interdental brush; DF: Dental floss, DFH: Dental floss holder

**Appendix S2-B.** Overview of **Plaque Index scores after single use** of the selected studies with various indices and their modifications. Baseline, end measurements and differences are presented as the (adjusted) means and standard deviations (SD) shown in parentheses. Statistically significant changes within groups are presented.

| **#**  **Authors (year)** | **Indices** | **Surfaces** | **Groups** | **Mean (SD)** | | | **Significant**  **within groups** |
| --- | --- | --- | --- | --- | --- | --- | --- |
|  |  |  |  | **Baseline** | **End** | **Difference** |  |
| **I**  **Yost**  **et al (2006)** | Benson modification (1993) of the Quigley & Hein (1962) Plaque Index | All | RBIC  IDB | 1.95 (?)  1.84 (?) | 1.44 (?)  1.29 (?) | -0.51◊ (?)  -0.55◊ (?) | yes  yes |
|  |  |  | RBIC  DF | 1.95 (?)  2.06 (?) | 1.44 (?)  1.51 (?) | -0.51◊ (?)  -0.55◊ (?) | yes  yes |
|  |  |  | RBIC  DFH | 1.95 (?)  1.98 (?) | 1.44 (?)  1.36 (?) | -0.51◊ (?)  -0.62◊ (?) | yes  yes |
| **II**  **Abouassi**  **et al (2014)** | Turesky (1970) modification of the Quigley & Hein (1962) Plaque Index | Accessible | RBIC  IDB | 2.02 (0.47♦)  2.00 (0.47♦) | 1.58 (0.33♦)  1.47 (0.35♦) | -0.44♦ (0.03♦)  -0.54♦ (0.03♦) | yes  yes |

?: unknown; ◊: calculated by the authors of this review based on the presented data in the selected paper; ♦: obtained by the original authors; SD: standard deviation

RBIC: Rubber bristle interdental cleaner; IDB: Interdental brush; DF: Dental floss, DFH: Dental floss holder

**Appendix S2-C.** Overview of **Bleeding Scores** of the selected studies with various indices and their modifications. Baseline, end measurements and differences are presented as the (adjusted) means and standard deviations (SD) shown in parentheses. Statistically significant changes within groups are presented.

| **#**  **Authors (year)** | **Indices** | **Surfaces** | **Groups** | **Mean (SD)** | | | **Significant**  **within groups** |
| --- | --- | --- | --- | --- | --- | --- | --- |
|  |  |  |  | **Baseline** | **End** | **Difference** |  |
| **I**  **Yost**  **et al (2006)** | Eastman Interdental Bleeding index  Caton & Polson (1985) | All | RBIC  IDB | 0.79 (?)  0.64 (?) | 0.26 (?)  0.14 (?) | 0.53 (?)  0.50 (?) | yes  yes |
|  |  |  | RBIC  DF | 0.79 (?)  0.58 (?) | 0.26 (?)  0.23 (?) | 0.53 (?)  0.36 (?) | yes  yes |
|  |  |  | RBIC  DFH | 0.79 (?)  0.73 (?) | 0.26 (?)  0.32◊ (?) | 0.53 (?)  0.41 (?) | yes  yes |
| **II**  **Abouassi**  **et al (2014)** | Eastman Interdental Bleeding index  Caton & Polson (1985) | Accessible | RBIC  IDB | 0.31 (0.14♦)  0.29 (0.13♦) | 0.06 (0.06♦)  0.09 (0.08♦) | -0.24♦ (0.01♦)  -0.21♦ (0.01♦) | yes  yes |
| **III**  **Hennequin-Hoenderdos**  **et al (2017)** | Bleeding On Marginal Probing Van der Weijden et al (1994) | All | RBIC  IDB | 1.09 (0.39)  1.07 (0.42) | 0.45 (0.25)  0.52 (0.25) | -0.64◊ (?)  -0.55◊ (?) | ?  ? |
|  |  | Accessible  mesial & distal | RBIC  IDB | 1.04 (0.48)  1.00 (0.47) | 0.34 (0.24)  0.45 (0.26) | -0.70◊ (?)  -0.55◊ (?) | ?  ? |
| **IV**  **Graziani**  **et al (2018)** | Bleeding On Marginal Probing/ Angular Bleeding index Van der Weijden et al (1994) | All | RBIC  TB | 0.12♦ (0.84♦)  0.19 ♦ (0.13♦) | 0.04♦ (0.05♦)  0.12 ♦ (0.11♦) | -0.08♦ (0.10♦)  -0.07 ♦ (0.09 ♦) | ?  ? |
|  |  |  | RBIC  IDB | 0.12♦ (0.84♦)  0.10♦ (0.08♦) | 0.04♦ (0.05♦)  0.05 ♦ (0.07♦) | -0.08♦ (0.10♦)  -0.05 ♦ (0.12♦) | ?  ? |
|  |  |  | RBIC  DF | 0.12♦ (0.84♦)  0.14♦ (0.11♦) | 0.04♦ (0.05♦)  0.77♦ (0.09♦) | -0.08♦ (0.10♦)  -0.10 ♦ (0.14♦) | ?  ? |
|  |  | Interdental | RBIC  TB | 0.25♦ (0.25♦)  0.29♦ (0.20♦) | 0.09♦ (0.09♦)  0.16♦ (0.09♦) | -0.20♦ (0.24♦)  -0.13♦ (0.17♦) | ?  ? |
|  |  |  | RBIC  IDB | 0.25♦ (0.25♦)  0.29♦ (0.25♦) | 0.09♦ (0.09♦)  0.12♦ (0.11♦) | -0.20♦ (0.24♦)  -0.13♦ (0.22♦) | ?  ? |
|  |  |  | RBIC  DF | 0.25♦ (0.25♦)  0.38♦ (0.20♦) | 0.09♦ (0.09♦)  0.19♦ (0.11♦) | -0.20♦ (0.24♦)  -0.19♦ (0.17♦) | ?  ? |
|  | Bleeding score | All | RBIC  TB | 17.3% (16.6%)  18.4% (13.4%) | 8.5% (8.0%)  14.0% (7.7%) | -8.8%◊ (?)  -4.4%◊ (?) | no  no |
|  |  |  | RBIC  IDB | 17.3% (16.6%)  14.4% (11.5%) | 8.5% (8.0%)  10.0% (6.6%) | -8.8%◊ (?)  -4.4%◊ (?) | no  no |
|  |  |  | RBIC  DF | 17.3% (16.6%)  22.3% (12.6%) | 8.5% (8.0%)  12.3% (7.2%) | -8.8%◊ (?)  -10.0%◊ (?) | no  yes |
|  |  | Interdental | RBIC  TB | 22.5% (22.3%)  20.7% (16.0%) | 12.1% (10.8%)  18.8% (11.3%) | -10.4%◊ (?)  -1.9%◊ (?) | no  no |
|  |  |  | RBIC  IDB | 22.5% (22.3%)  24.1% (27.4%) | 12.1% (10.8%)  13.9% (12.5%) | -10.4%◊ (?)  -10.2%◊ (?) | no  no |
|  |  |  | RBIC  DF | 22.5% (22.3%)  33.2% (20.8%) | 12.1% (10.8%)  22.1% (13.1%) | -10.4%◊ (?)  -11.1%◊ (?) | no  yes |
| **V**  **Moretti**  **et al (2020)** | Bleeding on probing | All | RBIC  DF | 15.7% (17.2%)  13.0% (12.7%) | 21.7% (16.6%)  26.6% (18.0%) | +7.0% (21.2%)  +13.0% (17.7%) | no  yes |
| **VI**  **Ustaoğlu**  **et al (2020)** | Papillary Bleeding Index (PBI) Saxer & Muehlemann (1975) | All | RBIC  IDB | 2.53 (0.52)  2.46 (0.47) | 0.49 (0.18)  0.45 (0.21) | -2.04◊ (?)  -2.01◊ (?) | Yes  yes |

?: unknown; ◊: calculated by the authors of this review based on the presented data in the selected paper; ♦: obtained by the original authors; SD: standard deviation

RBIC: Rubber bristle interdental cleaner; IDB: Interdental brush; DF: Dental floss, DFH: Dental floss holder

**Appendix S2-D.** Overview of **Gingival Index scores** of the selected studies with various indices and their modifications. Baseline, end measurements and differences are presented as the (adjusted) means and standard deviations (SD) shown in parentheses. Statistically significant changes within groups are presented.

| **#**  **Authors (year)** | **Indices** | **Surfaces** | **Groups** | **Mean (SD)** | | | **Significant**  **within groups** |
| --- | --- | --- | --- | --- | --- | --- | --- |
|  |  |  |  | **Baseline** | **End** | **Difference** |  |
| **I**  **Yost**  **et al (2006)** | Gingival Index Löe & Silness (1963) | All | RBIC  IDB | 1.31 (?)  1.38 (?) | 0.88 (?)  0.78 (?) | -0.43◊ (?)  -0.60◊ (?) | yes  yes |
|  |  |  | RBIC  DF | 1.31 (?)  1.36 (?) | 0.88 (?)  0.95 (?) | -0.43◊ (?)  -0.41◊ (?) | yes  yes |
|  |  |  | RBIC  DFH | 1.31 (?)  1.35 (?) | 0.88 (?)  0.91 (?) | -0.43◊ (?)  -0.44◊ (?) | yes  yes |
|  |  |  | RBIC  DFH | 1.46 (?)  1.47 (?) | 1.02 (?)  1.07 (?) | -0.44 (?)  -0.39 (?) | yes  yes |
| **V**  **Moretti**  **et al (2020)** | Gingival Index Löe & Silness (1963) | Accessible | RBIC  DF | 0.63 (0.24)  0.59 (0.34) | 0.61 (0.31)  0.62 (0.31) | -0.02 (0.35)  +0.03 (0.37) | no  no |

?: unknown; ◊: calculated by the authors of this review based on the presented data in the selected paper; ♦: obtained by the original authors; SD: standard deviation

RBIC: Rubber bristle interdental cleaner; IDB: Interdental brush; DF: Dental floss, DFH: Dental floss holder++

**Appendix S2-E.** Overview of **Gingival Abrasion scores** of the selected studies with various indices and their modifications. Baseline, end measurements and differences are presented as the (adjusted) means and standard deviations (SD) shown in parentheses. Statistically significant changes within groups are presented.

| **#**  **Authors (year)** | **Indices** | **Groups** | **Mean (SD)** | | | **Significant**  **within groups** |
| --- | --- | --- | --- | --- | --- | --- |
|  |  |  | **Baseline** | **End** | **Difference** |  |
| **III**  **Hennequin-Hoenderdos**  **et al (2017)** | Gingival Abrasion score  Van der Weijden (2004) all surfaces & sizes | RBIC  IDB | 5.10 (2.69)  5.57 (3.10) | 11.50 (4.04)  14.81 (4.35) | +6.40◊ (?)  +9.24◊ (?) | ?  ? |

?: unknown; ◊: calculated by the authors of this review based on the presented data in the selected paper; ♦: obtained by the original authors; SD: standard deviation

RBIC: Rubber bristle interdental cleaner; IDB: Interdental brush; DF: Dental floss, DFH: Dental floss holder

**Appendix S2-F.** Overview of **Panellist Preference assessment** of the selected studies.

| **#**  **Authors (year)** | **Indices** | **Question** | **Scale** |  | **Mean (SD)** | | |
| --- | --- | --- | --- | --- | --- | --- | --- |
|  |  |  |  | **RBIC** | **IDB** | **Difference** | **p-value**  **[95% CI]** |
| **II**  **Abouassi**  **et al (2014)** | 1-5 Likert score | Overall assessment | 1 very satisfied  5 very unsatisfied | 1.69 (0.79) | 2.35 (0.89) | 0.66◊ (?) | <0.01  [?] |
|  |  | Cleaning capacity | 1 very satisfied  5 very unsatisfied | 1.92 (0.91) | 2.27 (0.87) | 0.35◊ (?) | <0.05  [?] |
| **III**  **Hennequin-Hoenderdos**  **et al (2017)** | 0-10 VAS score | I found the use | 0 very unpleasant  10 very pleasant | 7.77 (1.69) | 4.48 (2.64) | 3.28 (2.94) | 0.000  [2.36; 4.20] |
|  |  | I found it scary to use | 0 I do not agree  10 I fully agree | 0.87 (1.42) | 2.58 (3.04) | 1.72 (3.10) | 0.001  [-2.69; -0.76] |
|  |  | The spaces between my teeth are cleaned well | 0 I do not agree  10 I fully agree | 7.37 (2.32) | 7.08 (2.23) | 0.29 (3.49) | 0.596  [-0.80; 1.37] |
|  |  | I had the feeling it damaged my gums | 0 I do not agree  10 I fully agree | 2.22 (2.80) | 5.94 (2.67) | 3.28 (2.67) | 0.000  [-4.11; -2.45] |
| **VI**  **Ustaoğlu**  **et al (2020)** | 1-5 Likert score♦ | Comfort | 1 very satisfied  5 very unsatisfied | 2 | 3 | - | 0.001 |
|  |  | Pain | 1 no Pain  10 worst Pain | 0 | 0 | - | 0.002 |
|  |  | Overall assessment | 1 very satisfied  5 very unsatisfied | 2 | 3 | - | 0.001 |
|  |  |  |  | **RBIC** | **DF** |  |  |
| **V**  **Moretti**  **et al (2020)** | 1-5 Rating Scale | Ease of use | 1 not easy to use  5 very easy to use | 3.80 (0.21) | 3.37 (0.17) | 0.43◊ (?) | <0.001 |
|  |  | Level of satisfaction | 1 not satisfied  5 very satisfied | 3.47 (0.16) | 3.76 (0.15) | 0.29◊ (?) | <0.001 |

?: unknown; ◊: calculated by the authors of this review based on the presented data in the selected paper; SD: standard deviation

RBIC: Rubber bristle interdental cleaner; IDB: Interdental brush, ♦: Median

**Appendix S3.** PRISMA checklist.

| **Section/topic** | **#** | **Checklist item** | **Reported on page #** |
| --- | --- | --- | --- |
| **TITLE** | | |  |
| Title | 1 | Identify the report as a systematic review, meta-analysis, or both. | 1 |
| **ABSTRACT** | | |  |
| Structured summary | 2 | Provide a structured summary including, as applicable: background; objectives; data sources; study eligibility criteria, participants, and interventions; study appraisal and synthesis methods; results; limitations; conclusions and implications of key findings; systematic review registration number. | 4 |
| **INTRODUCTION** | | |  |
| Rationale | 3 | Describe the rationale for the review in the context of what is already known. | 6-7 |
| Objectives | 4 | Provide an explicit statement of questions being addressed with reference to participants, interventions, comparisons, outcomes, and study design (PICOS). | 6-7 |
| **METHODS** | | |  |
| Protocol and registration | 5 | Indicate if a review protocol exists, if and where it can be accessed (e.g., Web address), and, if available, provide registration information including registration number. | 8 |
| Eligibility criteria | 6 | Specify study characteristics (e.g., PICOS, length of follow-up) and report characteristics (e.g., years considered, language, publication status) used as criteria for eligibility, giving rationale. | 8-9 |
| Information sources | 7 | Describe all information sources (e.g., databases with dates of coverage, contact with study authors to identify additional studies) in the search and date last searched. | 8 |
| Search | 8 | Present full electronic search strategy for at least one database, including any limits used, such that it could be repeated. | Table 1 |
| Study selection | 9 | State the process for selecting studies (i.e., screening, eligibility, included in systematic review, and, if applicable, included in the meta-analysis). | 8-9 |
| Data collection process | 10 | Describe method of data extraction from reports (e.g., piloted forms, independently, in duplicate) and any processes for obtaining and confirming data from investigators. | 10 |
| Data items | 11 | List and define all variables for which data were sought (e.g., PICOS, funding sources) and any assumptions and simplifications made. | 10 |
| Risk of bias in individual studies | 12 | Describe methods used for assessing risk of bias of individual studies (including specification of whether this was done at the study or outcome level), and how this information is to be used in any data synthesis. | 9 |
| Summary measures | 13 | State the principal summary measures (e.g., risk ratio, difference in means). | 9-10 |
| Synthesis of results | 14 | Describe the methods of handling data and combining results of studies, if done, including measures of consistency (e.g., I^2^) for each meta-analysis. | 10 |
| Risk of bias across studies | 15 | Specify any assessment of risk of bias that may affect the cumulative evidence (e.g., publication bias, selective reporting within studies). | 9-11 |
| Additional analyses | 16 | Describe methods of additional analyses (e.g., sensitivity or subgroup analyses, meta-regression), if done, indicating which were pre-specified. | 9-11 |
| **RESULTS** | | |  |
| Study selection | 17 | Give numbers of studies screened, assessed for eligibility, and included in the review, with reasons for exclusions at each stage, ideally with a flow diagram. | Figure 1 |
| Study characteristics | 18 | For each study, present characteristics for which data were extracted (e.g., study size, PICOS, follow-up period) and provide the citations. | Table 2 |
| Risk of bias within studies | 19 | Present data on risk of bias of each study and, if available, any outcome level assessment (see item 12). | S1 |
| Results of individual studies | 20 | For all outcomes considered (benefits or harms), present, for each study: (a) simple summary data for each intervention group (b) effect estimates and confidence intervals, ideally with a forest plot. | S2 |
| Synthesis of results | 21 | Present results of each meta-analysis done, including confidence intervals and measures of consistency. | NA |
| Risk of bias across studies | 22 | Present results of any assessment of risk of bias across studies (see Item 15). | S1 |
| Additional analysis | 23 | Give results of additional analyses, if done (e.g., sensitivity or subgroup analyses, meta-regression [see Item 16]). | 14-16 |
| **DISCUSSION** | | |  |
| Summary of evidence | 24 | Summarize the main findings including the strength of evidence for each main outcome; consider their relevance to key groups (e.g., healthcare providers, users, and policy makers). | 15-16 |
| Limitations | 25 | Discuss limitations at study and outcome level (e.g., risk of bias), and at review-level (e.g., incomplete retrieval of identified research, reporting bias). | 15-16 |
| Conclusions | 26 | Provide a general interpretation of the results in the context of other evidence, and implications for future research. | 15-16 |
| **FUNDING** | | |  |
| Funding | 27 | Describe sources of funding for the systematic review and other support (e.g., supply of data); role of funders for the systematic review. | 15-16 |

**Appendix S4.** References online appendixes.

Ainamo J, Bay I. Problems and proposals for recording gingivitis and plaque. *Int Dent J*. 1975;25(4):229-35.

Benson BJ, Henyon G, Grossman E, Mankodi S, Sharma NC. Development and verification of the proximal/marginal plaque index. *The Journal of clinical dentistry*.

1993;4(1):14-20.

Caton JG, Polson AM. The interdental bleeding index: a simplified procedure for monitoring gingival health. *Compendium of continuing education in dentistry*. 1985;6(2):88, 90-2.

Lobene RR, Soparkar PM, Newman MB. Use of dental floss. Effect on plaque and gingivitis. *Clinical preventive dentistry*. 1982;4(1):5-8.

Loe H, Silness J. PERIODONTAL DISEASE IN PREGNANCY. I. PREVALENCE AND SEVERITY. *Acta odontologica Scandinavica*. 1963;21:533-51.

O'Leary TJ, Drake RB, Naylor JE. The plaque control record. *The Journal of periodontology*. 1972;43(1):38.

gingivitis. *Journal of clinical periodontology*. 1994;21(9):589-94.

Quigley GA, Hein JW. Comparative cleansing efficiency of manual and power brushing. *J Am Dent Assoc*. 1962;65:26-9.

Saxer UP, Mühlemann HR. Motivation und Aufklärung [Motivation and education]. SSO Schweiz Monatsschr Zahnheilkd. 1975;85(9):905-919.

Turesky S, Gilmore ND, Glickman I. Reduced plaque formation by the chloromethyl analogue of victamine C. *The Journal of periodontology*. 1970;41(1):41-3.

Van der Weijden GA, Timmerman MF, Nijboer A, Reijerse E, Van der Velden U. Comparison of different approaches to assess bleeding on probing as indicators of
